# Establishing control and breaking transmission: the importance of accurately classifying cure following treatment for soil-transmitted helminths

**DOI:** 10.1016/j.lanwpc.2021.100288

**Published:** 2021-10-21

**Authors:** Judd L Walson

**Affiliations:** Departments of Global Health, Medicine, Pediatrics and Epidemiology, University of Washington, Seattle, WA, USA

Soil-transmitted helminth infections (STH) remain among the most common pathogens infecting humans globally [1]. Cumulatively, these infections result in an estimated disease burden of 3.39 million disability adjusted life years annually [2]. In particular, high-intensity infections are associated with significant morbidity, particularly among children and pregnant women. Over the past several decades, deworming programs have adopted a high successful morbidity control strategy targeting empiric treatment of populations at highest risk of morbidity. These programs are largely supported by the generous donation of anthelmintic drugs from GlaxoSmithKline (albendazole) and Johnson & Johnson (mebendazole). As a result of these programs, estimates suggest that as much as 75% if all morbidity due to STH infections may be averted if current coverage targets are reached.

However, because these programs are designed to target only populations at high risk of morbidity, a large reservoir of infection persists in untreated adults and non-school going children. There are important implications that result from these persistent reservoirs of infection. In many settings, STH control programs will need to be maintained indefinitely, or at least until substantial reductions in poverty and improvements in infrastructure are achieved. In addition, microscopy-based diagnostics currently utilized by STH programs are highly insensitive, particularly for the detection of low-intensity STH infections. The combination of continued transmission of infection within communities, sustained drug pressure from repeated mass drug administration and a failure of current diagnostics to accurately identify infection poses substantial risks to population health.

In a recent issue of *The Lancet Region Health – Western Pacific*, Colella and colleagues describe the results of a survey conducted across ten villages in Cambodia in which samples were collected to ascertain hookworm infection prevalence [3]. While the results of the prevalence survey are limited by the sampling methodology, the data on cure rates is informative and of relevance to public health programs. Among individuals who were found to be positive for hookworm by standard faecal flotation (SFF) methods, additional follow up samples were collected at least one weak following treatment with albendazole to determine cure rates. The cure rate demonstrated by SFF in this study (81.5%) was in line with previous studies that have documented cure rates with albendazole. For example, a systematic review of studies evaluating cure rates in randomized controlled trials documented cure rates with albendazole for hookworm spp. of 79.5% (95% CI 71.5-85.6%) [4]. However, when evaluated using qPCR, the cure rate following treatment with albendazole in this study was substantially lower (46.4%).

These results demonstrate that cure rates are dramatically overestimated when relying on microscopy-based diagnostics. Other studies have also determined that the risk of misclassification due to poor diagnostic performance of microscopy-based diagnostics is greatest for hookworm spp [5]. In addition, the sensitivity of these microscopy-based assays decreases as the burden of infection is reduced [6]. As a result, the use of microscopy to estimate infection following treatment, when burden is expected to be dramatically reduced, is likely to result in a dramatic overestimation of cure rate, as low-intensity infections will be preferentially missed using microscopy. The implications of these findings are of significant public health importance, as surveillance efforts within ongoing programs are likely to increasingly misclassify infection prevalence following repeated rounds of treatment. This may lead to premature cessation of program activities or to changes to the planned frequency of future treatment. In addition, the inability to detect substantial numbers of infection among populations in whom drug pressure is significant may lead to the failure to observe meaningful changes in treatment efficacy due to the emergence of resistance. Benzimidazole resistance has been shown to rapidly emerge in animal populations with repeated treatment and as STH programs increasingly scale up treatment frequency and intensity, the risk of such resistance developing in human populations may be substantial [7]. Early detection of resistance emergence is critical to prevent the wide-spread failure of treatment observed in other pathogens with the emergence of resistance.

Finally, the failure to correctly identify infection in treated populations may have important consequences for decisions related to stopping STH treatment programs. Increasingly, there is interest in identifying strategies to interrupt transmission using alternative anthelmintic treatment strategies. Mathematic models suggest that transmission interruption may be possible in some geographic settings by treating entire communities with multiple rounds of anthelmintic drugs at high coverage and compliance [8]. In addition, a number of large population-based studies are now being conducted to evaluate the feasibility of interrupting STH transmission through intensive community-wide MDA [9,10]. If and when programs move towards transmission interruption, the continued use of diagnostics that misclassify infection status threatens the ability to determine if or when the threshold for transmission interruption has occurred. Novel molecular diagnostic techniques appear to be critical to measuring the impact of treatment and determining when transmission interruption has been reached.

## Author Contributions

JLW conceived of and wrote all content included in this commentary.

## Declarations of Interest

I have received grant funding from the Bill & Melinda Gates Foundation for work related to STH transmission interruption and to the development of qPCR based methods for STH identification.

## Declaration of Competing Interest

The authors declare no conflict of interest.
